# *In situ* microradioscopy and microtomography of fatigue-loaded dental two-piece implants

**DOI:** 10.1107/S1600577515015763

**Published:** 2015-10-09

**Authors:** Wolfram Wiest, Simon Zabler, Alexander Rack, Christian Fella, Andreas Balles, Katja Nelson, Rainer Schmelzeisen, Randolf Hanke

**Affiliations:** a Institute of Physics, University of Würzburg, Germany; b European Synchrotron Radiation Facility, France; c Department of Oral and Maxillofacial Surgery, Medical Centre – University of Freiburg, Germany; d Fraunhofer EZRT, Fürth, Germany

**Keywords:** *in situ* radioscopy, microtomography, dental implants, fatigue

## Abstract

Synchrotron real-time radioscopy and *in situ* microtomography are the only techniques providing direct visible information on a micrometre scale of local deformation in the implant–abutment connection (IAC) during and after cyclic loading. The microgap formation at the IAC has been subject to a number of studies as it has been proposed to be associated with long-term implant success. The next step in this scientific development is to focus on the *in situ* fatigue procedure of two-component dental implants. Therefore, an apparatus has been developed which is optimized for the *in situ* fatigue analysis of dental implants. This report demonstrates both the capability of *in situ* radioscopy and microtomography at the ID19 beamline for the study of cyclic deformation in dental implants. The first results show that it is possible to visualize fatigue loading of dental implants in real-time radioscopy in addition to the *in situ* fatigue tomography. For the latter, *in situ* microtomography is applied during the cyclic loading cycles in order to visualize the opening of the IAC microgap. These results concur with previous *ex situ* studies on similar systems. The setup allows for easily increasing the bending force, to simulate different chewing situations, and is, therefore, a versatile tool for examining the fatigue processes of dental implants and possibly other specimens.

## Abstract 2

Results of a novel *in situ* microradiography and microtomography setup for the study of fatigue processes are presented. This setup is optimized for the requirements of dental implants and use at synchrotron imaging beamlines.

## 1. Introduction   

Most taper implant–abutment connections show a higher fatigue resistance when compared with butt-joint connections but their maximum load bearing capacity is similar with approximately 400 N until failure (Dittmer *et al.*, 2011 ▸, 2012 ▸). High-cycle fatigue (HCF) with low loading forces up to 120 N revealed the occurrence of a plastic deformation of the implant-shoulder and within the implant–abutment interface (Rack *et al.*, 2013 ▸; Wiest *et al.*, 2015 ▸; Nelson *et al.*, 2015 ▸). It is known that occlusal forces can amount up to 1000 N with a mean maximum biting force of 578 N at the implant-supported prosthesis side (Shinkai *et al.*, 2007 ▸; Al-Omiri *et al.*, 2014 ▸).

The existence and increase in microgap formation after fatigue loading have been reported independent of the implant–abutment connection (IAC) design and are thought to be involved in the occurrence of peri-implantitis (Harder *et al.*, 2010 ▸). The etiology of peri-implantitis is associated with the existence of bacteria in and around the implant (Klinge & Meyle, 2012 ▸). Peri-implantitis, with a prevalence of 22%, occurs in implants independent of the implant–abutment design (Mombelli *et al.*, 2012 ▸). Recent work on fatigue deformation in tapered two-piece dental titanium implants has revealed microdeformations along the IAC, well below the fatigue limit (Zabler *et al.*, 2012 ▸). These deformations may lead to a loss in mechanical stability of the tooth replacement and, in the worst case, can cause implant loosening or fracture (Gomes *et al.*, 1996 ▸; Harder *et al.*, 2010 ▸). At the high-cycle fatigue limit (10^6^ cycles), *ex situ* synchrotron microtomography has revealed this increase in abutment mobility and microgap size upon extra-axial load yet the low-cycle fatigue (LCF) limit has not been explored yet (Rack *et al.*, 2013 ▸; Wiest *et al.*, 2015 ▸). The reason for the lack of measurements on LCF deformation in tapered implants lies within the limited availability of *in situ* X-ray microscopy techniques, both in two and in three dimensions.

*In situ* microradioscopy and microtomography for the study of LCF deformation require frame rates of the order of 20 Hz to 150 Hz in order to image a single-shot image acquisition without motion artefacts at the crest of one fatigue cycle and without the need to accumulate the photons in a stroboscopic mode. In addition to the micrometre resolution and the high photon flux at ∼60 keV mean energy, the ID19 beamline provides a quasi-parallel beam which makes the use of the inline phase contrast an especially simple option for imaging the smallest features within a sample. The challenge is to find an optimum between high flux at approximately 60 keV for fast X-ray imaging of titanium probes and a sufficiently narrow bandwidth for the inline phase contrast. Fast imaging is essentially due to the short exposure time of a few milliseconds, for reaching quasi-stationary conditions at force maximum with a force repetition rate of 10 Hz. Only synchrotron beamlines which are operated in a narrow polychromatic wiggler spectrum mode (so-called pink beam) allow for these conditions. Measuring microgaps which form at the IAC in two-piece implants under extra-axial load make the use of phase contrast imaging compulsory: unless the IAC gap is 20 µm or larger it can only be detected from high-resolution phase contrast images. In simple X-ray absorption images it is difficult to separate objects of similar material. With the edge enhancement of inline phase contrast (Mayo *et al.*, 2012 ▸) it becomes possible to identify even weak interfaces (Snigirev *et al.*, 1995 ▸; Cloetens *et al.*, 1996 ▸). By using this technique we have demonstrated that microgaps down to 100 nm width can be measured in two and three dimensions; in the latter case, the microgap at the IAC can be unwrapped onto a two-dimensional map which shows the local gap width (Zabler *et al.*, 2012 ▸).

Maire & Withers (2014 ▸) pointed out in their review that many communities are interested in *in situ* fatigue analysis, which is easier to perform at synchrotron sources due to high flux. To our best knowledge, *in situ* fatigue tomographies are mostly performed after cyclic treatment, similar to the work of Bleuet *et al.* (2004 ▸). As the interest for understanding fatigue processes is high, the variety of *in situ* approaches is also high (Fischer *et al.*, 2013 ▸; Forsberg *et al.*, 2008 ▸; Hirano *et al.*, 1995 ▸; Müller *et al.*, 2006 ▸). Buffiere *et al.* (2010 ▸) carried out fatigue experiments on a tomographic sample stage using a rotating cam but the mechanical loading was stopped during the tomographic scans. Garcea *et al.* (2014 ▸) performed fatigue cycling initially in a standard fatigue machine; then the sample was dismounted for the tomographic measurements which were acquired afterwards with and without static loading. Our work demonstrates the capability of *in situ* two-dimensional and three-dimensional fatigue imaging during the running fatigue process in the pink beam configuration to measure LCF deformation in two-piece dental implants.

## 2. Materials and methods   

**Figure 1:**
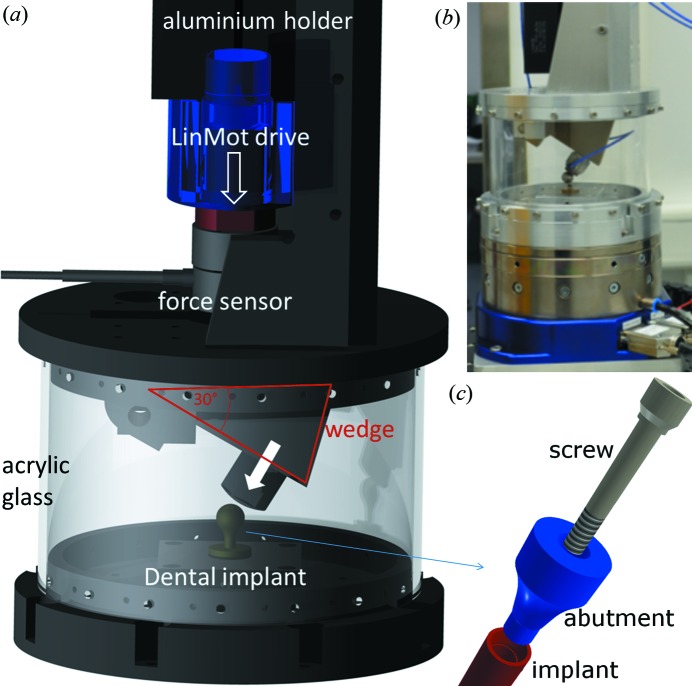
(*a*) Apparatus for *in situ* two-dimensional and three-dimensional fatigue testing on the ID19 beamline. Essential parts are labelled in the CAD-rendering. (*b*) Photograph of the setup. (*c*) Close-up view of a two-component dental implant. The implant (red) is inserted into the jawbone. The abutment (blue) is plugged into the implant and fixed with the screw (grey).

A new *in situ* dental implant testing device specially designed for the experiments was used and is detailed in Fig. 1 ▸. The main issue was to apply fatigue treatment simultaneously with microtomography at synchrotron beamlines. Therefore, the engine for force treatment needed to be placed above the specimen and, due to the rotary table, the testing device need to be mass centred. The underlying testing norm (DIN ISO EN 14801:2007) requires a force inclination of 30°. Therefore, the vertical motion is redirected by a metallic wedge to 30° (see Fig. 1 ▸). Its main component is, nevertheless, a linear direct drive (P01-48×360; NTI AG LinMot & MagSpring, Switzerland) which can apply up to 500 N cyclic extra axial (30°) forces with up to 10 Hz frequency. The drive comprises a motion control based on length encoder (strain-controlled fatigue) and a DC force sensor (8435, Burster GmbH, Germany) for stress-controlled fatigue. The latter mode was used in the present experiments. For reaching the time accuracy between the motion and the exact X-ray acquisition, the relevant parts are controlled by an FPGA system (Compact­RIO, National Instruments, USA).

The experiments were performed at the ESRF ID19 beamline in pink beam wiggler configuration at ∼60 keV. The sufficiently narrow bandwidth for the inline phase contrast was achieved by a combination of X-ray absorber (diamond, aluminium and tungsten). The study can be divided into two parts: first, real-time microradioscopy (µXR), and, second, *in situ* X-ray microtomography (µCT). µXR movies and µCT scans were recorded with a fast indirect X-ray camera which comprises the Dimax CMOS camera (PCO GmbH, Germany) and 4× magnifying optics which record the images from a LuAG:Eu screen (for tomography; for radiography a GGG screen was used instead) *via* a 45° mirror in order to avoid radiation damage to the CMOS sensor of the camera and the lenses. The effective pixel size was 2.75 µm (spatial resolution was at a maximum of the order 5.5 µm according to the Nyquist criterion) and the exposure times were 10 ms per frame for both µXR and µCT (2016 × 2016 pixel array).

First, for the radioscopy study, a cyclic load was applied under a 30° inclination with respect to the main implant axis, respecting the force ratio *R* = 0.1 with *F*_max_ = 500 N and *F*_min_ = 50 N and 1 Hz frequency. Thus ∼100 frames are recorded during one load cycle. The loaded implant was a BoneLevel (BL) (*D* = 3.3 mm, *L* = 12 mm; ref. No. 021.2312) commercially available two-piece implant from Straumann AG (Switzerland). The microgap at the IAC was calculated for each frame using a numerical method described by Zabler *et al.* (2010 ▸).

Second, for the *in situ* µCT measurement, the cyclic load was increased stepwise from 50, 100, 150, 200 to 250 N peak force. For each fatigue load, ten µCT scans were recorded, each from 1000 projection images, hence representing 10000 cycles. After changing the load, 5000 cycles were applied without recording, in order to give the system time to settle to the new force so that for the remaining 10000 cycles quasi-stationary conditions could be assumed. Hence, a total of 75000 cycles was applied to the sample, this time with 10 Hz frequency, thus shortening the exposure times to 10 ms per frame. The sample was a two-piece Astra OsseoSpeed TX (AS) implant (*D* = 3.0 mm, *L* = 11 mm; ref. No. 24982) (Dentsply, Germany). From the *in situ* µCT scans, the microgap was visualized in the form of cylindrical projection maps of the radial Fresnel fringe contrast along the IAC (see Zabler *et al.*, 2012 ▸), which has been shown to be proportional to the actual gap width.

**Figure 2:**
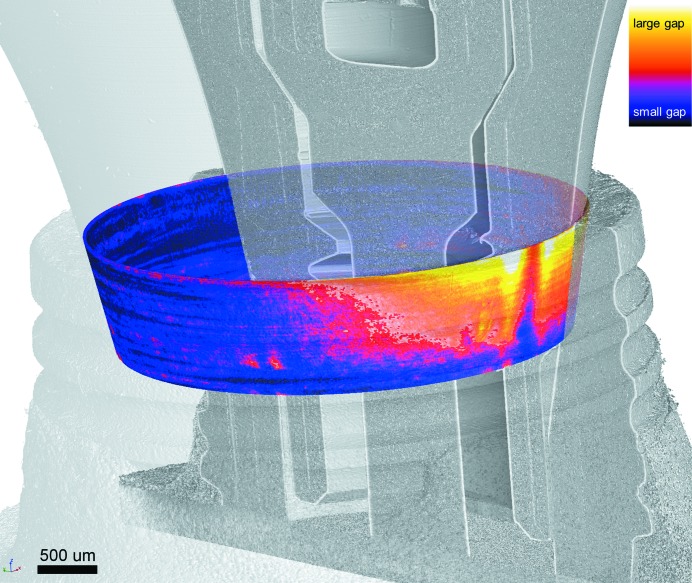
Exemplary three-dimensional visualization of an IAC microgap map (coloured) superimposed on the CT scan of the dental implant, pointing out the orientation of the latter. Here the implant is a NobelActive (Nobel Biocare, Switzerland) under 333 N cyclic load. The IAC map is calculated according to Zabler *et al.* (2012 ▸); the look-up table is comparable with Fig. 6 ▸.

For improved understanding of the used IAC map visualization (see Zabler *et al.*, 2012 ▸) and their orientation a three-dimensional rendering is given in Fig. 2 ▸. In this figure an exemplary dental two-piece implant (NobelActive, Nobel Biocare Holding AG, Switzerland) is recorded under cyclic load of 333 N.

All samples were embedded in autopolymerizing acrylic resin (Technovit® 4000, Heraeus Kulzer, Wehrheim, Germany) according to DIN ISO EN 14801:2007 for dental implant fatigue testing. For all samples, the embedding resin is contained in a brass ring which, in turn, is held in the fatigue machine with screws which helps to avoid notching.

Note that for each experiment in this report a different implant system was used: Straumann, Astra and Nobel. The used implants are two-piece titanium implant systems with a tapered IAC.

## 3. Results   

**Figure 3:**
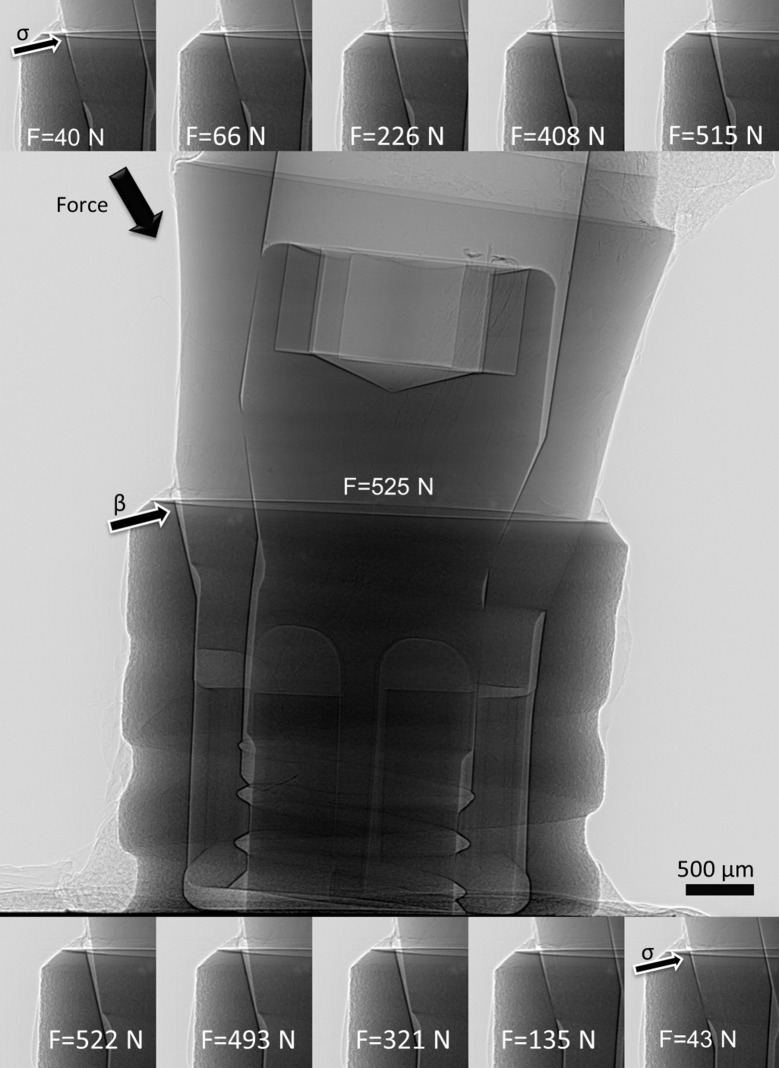
Representative frames from the real-time radioscopy experiment (middle maximum force). The gap opening at the implant shoulder is labelled with arrows, β indicates a big gap and σ indicates a small gap. The loaded implant was a BoneLevel (Straumann AG, Switzerland) commercially available two-piece implant. The full movie can be downloaded from the supporting information.

**Figure 4:**
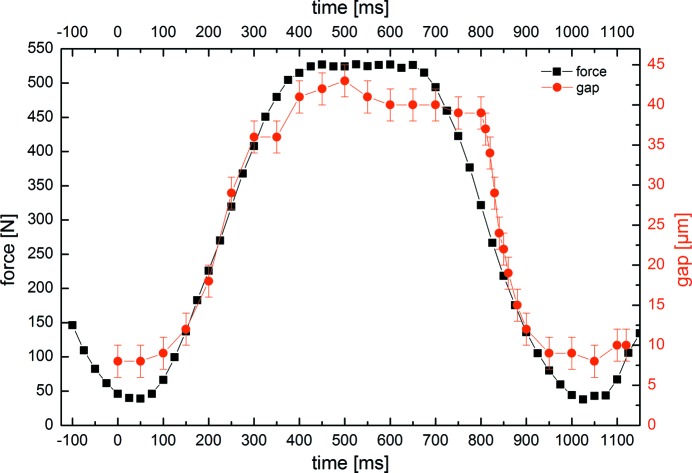
Measured force vector and calculated microgap for each time point for one cyclic deformation cycle (*ca* 1 Hz repeat). The gap values were calculated by the method detailed by Zabler *et al.* (2010 ▸). The error for the gap size is ±2 µm and the error for the measured force is an estimation of ±4 N.

The radioscopic movie of the BL implant system showing 100 frames during one loading cycle is given in the supporting information. Representative frames of the sequences are shown in Fig. 3 ▸. Note that an adequate number of cycles had been applied prior to this sequence in order to allow the system to settle. Besides a wide gap opening at the implant shoulder (arrow labelled β) adjacent to the oblique force application, the frames also show a significant residual gap at the minimum force values (40 N and 43 N, arrow labelled σ). Fig. 4 ▸ shows two curves: (i) the measured force values (in N) and (ii) the calculated microgap (in µm), both *versus* time (*ca* 1.2 s). Both curves correlate well, fostering the assumption that microgap opening and applied force are indeed connected *via* a linear elastic response. The measurements shown in Fig. 4 ▸ were repeated many times and found to reproduce minutely over various cycles.

**Figure 5:**
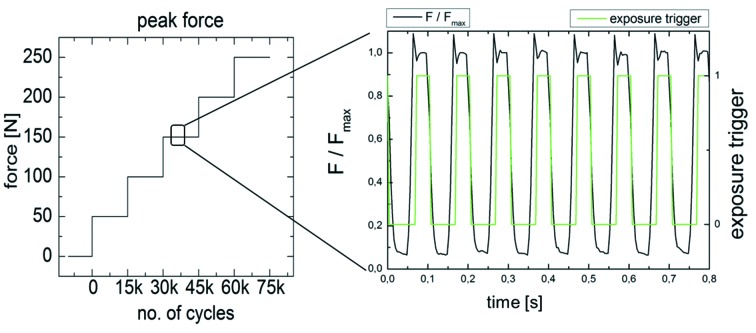
Application of increased cyclic load in steps of 50 N. For each force level 15000 cycles were applied, 5000 for setting and 10000 for recording ten *in situ* CT scans of the implant system. Each scan represents 1000 projection images (each 10 ms exposure) recorded over 180° incremental sample rotation. The rotation steps are triggered and performed in between two exposures.

**Figure 6:**
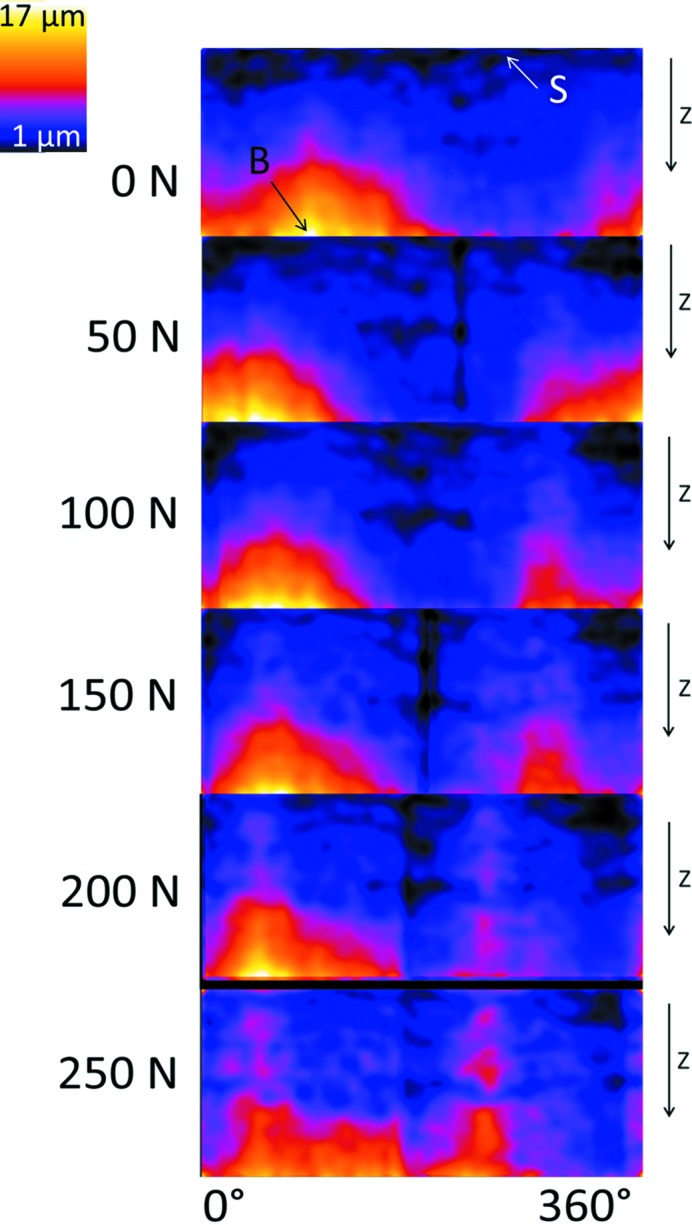
Sequence of cylindrical projection maps showing the local microgap at the IAC at each force level (the average of five microgap maps out of ten scans). Calculation of these maps was performed according to Zabler *et al.* (2012 ▸), bright values show larger and dark values a thinner microgap. Orientation is along the implant axis; for better understanding see Fig. 2 ▸. For a better understanding we calculate the exact gap size at two characteristic points (point B, 17 µm gap size; point S, 1 µm gap size; the error for the gap size is ±2 µm). At The sample was a two-piece implant Astra OsseoSpeed TX (Dentsply, Germany).

The applied cyclic force for the *in situ* µCT experiment is schematically shown in Fig. 5 ▸. For each load cycle (10 Hz), one radiographic image was triggered and exposed at the crest of the force (*F*_max_) with an exposure time of only 10 ms; the sample was then rotated by 0.18° with respect to the X-ray beam and the next exposure was triggered and so on until the 180° scan was complete with 1000 projection images. Altogether ten tomograms were recorded in each sequence (10000 cycles), then the force vector was increased by 50 N and the next sequence was triggered after 5000 initial cycles (altogether 15000 cycles per step). The resulting image quality was somewhat inferior to that shown in Fig. 3 ▸ due to a reduced photon flux. Nevertheless, data quality was sufficient for volume reconstruction. The assumption of the quasi-stationary conditions after 5000 initial cycles was sufficing as no motion artefacts were observable. From the three-dimensional volume images a projection method was used (see Zabler *et al.*, 2012 ▸) to project the fringe contrast with the gap width of the conical plane of the IAC. In addition, a Gaussian blur (50 pixel) was applied to the maps in order to compensate for the very low signal-to-noise ratio. The resulting IAC maps of the microgap width of the AS implant are shown for the average of five microgap maps for each force level in Fig. 6 ▸ along with the initial measurement (0 N) of the microgap prior to the fatigue. First, we note that a microgap is detected prior to fatigue in AS. It amounts to a 17 µm width at the lower end of the IAC (point B in Fig. 6 ▸). Upon force application there is a minor angular shift in the projection maps and the pattern at the IAC changes gradually with each force increase, resulting in an IAC map for the 250 N cyclic force which is visibly different (although less peaked) from the initial state of the sample. While the lower region of the IAC shows a gap which is slightly closed with increasing cyclic load, the upper half of the IAC starts to open up, mostly at two distinct angles which are 180° opposed to each other and which coincide with the direction of load application. No fracture was found in the AS implant after application of the 75000 cycles.

## 4. Discussion   

The real-time µXR measurements represent a very powerful tool for evaluating the elastic response under cyclic load for any two-piece implant system which is excited at a frequency of 1–2 Hz. In this part of our study the selected force amplitude was below the maximum biting force reported in the literature (Shinkai *et al.*, 2007 ▸; Al-Omiri *et al.*, 2014 ▸) but higher than the mean biting force initially reported for implants (Hattori *et al.*, 2009 ▸; Morneburg & Proschel, 2003 ▸) as we also wanted to demonstrate the technical abilities of our implant testing device and the response of the implant. From our previous studies and those of others, it is well known that, depending on the implant design and geometry, this elastic response can vary tremendously and, therefore, our method is a highly important marker for the mechanical comparison between different implant systems as well as for validating/optimizing finite-element analysis simulations. Fast cyclic *in situ* microtomography visualizes the opening of the microgap under cyclic bending load, which allows an almost realistic testing scenario. This technique is limited to synchrotron sources which outperform medical X-ray and other laboratory sources by many orders of magnitude in terms of high resolution at high acquisition rate. Our method further allows the validation and optimization of methods (*e.g.* finite-element analysis) to study the mechanical and, therefore, the clinical performance of dental implants.

The results from our *in situ* µCT measurements of the load increase experiment are in accordance with results extracted from previous *ex situ* studies on HCF-deformed implants of the same design (Zabler *et al.*, 2012 ▸; Rack *et al.*, 2013 ▸). Showing that the microgap of the IAC of this system (*a*) inverses the location of the gap (lower IAC instead of upper IAC) and (*b*) also tends to close the initial gap under static load, the result can now be extended to cyclic experiments. Increasing further the photon flux (*e.g.* by using more powerful insertion devices) will soon allow for such studies in higher detail (the IAC maps had to be averaged through a Gaussian filter which would become obsolete if the fringe signal was sufficiently strong) or a higher repetition rate (*e.g.* 100 Hz), thus permitting the study of HCF effects *in situ*. Our findings (two-dimensional) suggest that an entirely linear elastic response is taking place upon cyclic extra-axial load, yet it is known that residual microgaps and microdeformation of implants occur and remain after fatigue loading (Rack *et al.*, 2013 ▸; Nelson *et al.*, 2015 ▸). From this point we can conclude that this portion of plasticity must take place over very short timescales (first 1000 cycles or less), which are not imaged by our *in situ* µCT experiment. With increasing cyclic load the microgap in the AS implant appears to change to a thin opening along the entire IAC. Stepwise increase of the fatigue load produces microgap patterns at the IAC which are different from our previous *ex situ* observations, suggesting that a short pulse application of forces or a different pattern of fatigue-induced changes within the first 75000 cycles might lead to a different microgap formation. Nevertheless the existence of the microgap persists.

These preliminary results on the microscopic LCF deformation of dental two-piece implants suggest that there is a very high need for such direct insight into the micromechanics of these devices. Unlike static loading, the cyclic deformation of two-piece components is very difficult if not impossible to track by finite-element simulations. *In situ* synchrotron µCT during fatigue was pioneered by Bleuet *et al.* (2004 ▸). It has to be mentioned that the relations of IAC, crestal peri-implant bone loss (Passos *et al.*, 2013 ▸) and mechanical failure are discussed controversially. From our current point of view, we expect that the presented technique might even become a common tool in the design and development of new implants and materials. In addition to its use for dental implants, one might expand the field of application of our setup easily by changing the sample holder and the wedge. Hence, samples like fibre composites (Garcea *et al.*, 2014 ▸), wood or mechanical connections can be tested under cyclic fatigue load with respect to the characteristic performances of this testing device. Besides testing other specimens the apparatus could be developed further: one idea is to combine our setup with that presented by Fischer *et al.* (2013 ▸) so that high-cycle fatigue can be applied through an additional piezoelectric ultrasonic processor. During the µCT scan, the slower linear direct drive motor can be used. The benefit of such a two-stage fatigue setup is to access the high-cycle-fatigue range in the course of a synchrotron beam time.

## 5. Conclusions   

With our work, it could be successfully demonstrated that both (i) real-time radioscopy (µXR) of cyclic deformation in dental two-piece implants, as well as (ii) quasi-stationary *in situ* µCT scans (without stroboscopic averaging) are feasible at micrometre resolution including phase contrast mode, by using dedicated testing environments together with modern synchrotron light sources which are operated in pink beam configuration.

## Supplementary Material

Real-time radioscopy movie of force application on dental implant. The force varies from approximately 40 N to 525 N with a repetition rate of 1 Hz. The radiographies are recorded at 100 frames per second. For better visualisation this movie is played at 5 frames per second.. DOI: 10.1107/S1600577515015763/mo5115sup1.mp4
